# Case Report: Improvement Following Immunotherapy in an Individual With Seronegative Down Syndrome Disintegrative Disorder

**DOI:** 10.3389/fneur.2021.621637

**Published:** 2021-03-26

**Authors:** Sarah J. Hart, Gordon Worley, Priya S. Kishnani, Heather Van Mater

**Affiliations:** ^1^Department of Pediatrics, Division of Medical Genetics, Duke University Medical Center, Durham, NC, United States; ^2^Department of Pediatrics, Division of Pediatric Neurology, Duke University Medical Center, Durham, NC, United States; ^3^Department of Pediatrics, Division of Pediatric Rheumatology, Duke University Medical Center, Durham, NC, United States

**Keywords:** down syndrome, regression, autoimmunity, disintegrative disorder, IVIG

## Abstract

Down syndrome disintegrative disorder (DSDD) is a condition of unknown etiology characterized by acute cognitive decline, catatonia, insomnia, and autistic features in individuals with Down syndrome. A prior report of four patients with DSDD suggested a potential autoimmune etiology based on the presence of autoantibodies and on successful treatment with immunotherapy that included intravenous immunoglobulin (IVIG). Herein, we present the case of an 8-year old girl who developed acute cognitive decline to a dementia-like state, insomnia, catatonia, and autistic features. In contrast to the four patients with DSDD above, she had no evidence of autoimmunity and presented at a younger age. Given the gravity of her acute deterioration and the exclusion of other etiologies, she was treated with immunotherapy presumptively. She responded with near complete resolution of symptoms, but demonstrated a pattern of mild decline as she approached each monthly dosing of IVIG and steroids, reversed by treatment. Mycophenolate mofetil (MMF) was therefore added, with stability throughout the month and the ability to taper off IVIG. After stopping IVIG, she had a mild recurrence of symptoms that again resolved with repeat IVIG followed by tapering off. Outcome was assessed at 2.5 years after presentation, at which time she was back to her premorbid condition, except for persistent tics off immunotherapy. This case supports the contention that patients with a rapid onset of severe symptoms consistent with DSDD, who have a thorough evaluation with the exclusion of other etiologies, may warrant a trial of immunotherapy with steroids, IVIG and/or other agents like MMF even in the absence of evidence of autoimmunity on standard evaluation.

## Introduction

Down syndrome disintegrative disorder (DSDD) is a condition being increasingly recognized in individuals with Down syndrome (1, 2), associated with rapid onset of severe cognitive decline, features of catatonia, development of new or worsening features of autism spectrum disorder, sleep difficulty, and psychiatric symptoms (3, 4). DSDD has primarily been identified in adolescents and young adults with Down syndrome (4). While the entity of DSDD has been recognized across the Down syndrome medical community, there has been debate on the terminology to describe this condition with common terms including “regression”, “unexplained regression”, and “catatonia” in Down syndrome (4–7). The etiology of this condition is also unknown, with some hypothesized causes including psychiatric disorders, untreated obstructive sleep apnea, and environmental stressors, and autoimmunity (4, 5, 8–10).

Worley et al. used the term DSDD to describe a cohort of patients with Down syndrome and cognitive decline who also were found to have thyroid autoantibodies, suggesting that autoimmunity may be a possible cause of this condition (3). Individuals with Down syndrome have a higher risk for autoimmune disease compared to the general population, including Hashimoto's thyroiditis, celiac disease, alopecia areata, vitiligo, and inflammatory arthropathy. We previously reported a case series showing that immunotherapy led to significant improvements in symptoms of catatonia, autistic features, insomnia, cognition and psychosis in four individuals with DSDD, all of whom had some evidence of autoimmunity (8). Recent reviews have described the potential role of autoimmunity as an etiology for DSDD (1) and have noted the potential of immunotherapy as one of several possible treatment options (2).

We provide evidence in this case presentation for efficacy of immunotherapy in an individual with DSDD and no known evidence for autoimmunity. Records from over 1.5 years of clinical follow up were reviewed systematically and retrospectively according to the domains previously investigated by Cardinale et al. (8), with final outcome assessed at 2.5 years after presentation. These included features of autism spectrum disorder according to DSM-5 criteria (11), catatonia, insomnia, psychiatric symptoms, cognitive-behavioral characteristics, and neurological symptoms. Status at last follow up was based on information from her local provider. This case was determined as exempt by the Duke University Medical Center Institutional Review board. The patient's parents provided written informed consent for this publication.

## Case Report

An 8-year-old Caucasian girl with Down syndrome presented to the Duke pediatric autoimmune brain disease clinic after a single episode of disintegration with rapid catatonic presentation. Prior to her clinical deterioration, she had no features of autism spectrum disorder, catatonia, insomnia, psychiatric symptoms or neurologic symptoms. She was learning to read and could carry on back-and-forth conversations. She was not ill prior to her clinical deterioration. Her sister had strep pharyngitis several months prior to her deterioration, but the patient had no symptoms of strep pharyngitis herself.

She experienced an acute decline characterized by onset of catatonia, including immobility and non-responsiveness to environmental cues. She exhibited mutism with loss of word use and cessation of attempting to communicate. She spoke no more than one or two words over a period of days to weeks, with rare use of phrases or sentences. She exhibited staring for long periods even in the setting of others attempting to interact and posturing with standing or sitting in the same spot for hours. She began engaging in frequent motor stereotypies including clapping, rocking on her feet, and bringing her arms and legs to the midline repeatedly. She met diagnostic criteria for catatonia by her immobility, stereotypies, mutism, and unresponsiveness.

She developed features within all domains of autism spectrum disorder as defined by the DSM-5. Multiple deficits in social/emotional reciprocity were noted including abnormal social approach, reduced sharing of interests and overall loss of communication. Deficits in non-verbal communicative behaviors included poorly integrated verbal and non-verbal communication, loss of eye contact, deficits in use of gestures, and lack of facial expression with staring “past” others. Deficits in relationships included difficulties in sharing imaginative play and absence of interest in peers.

She developed sleep difficulty with sleep induction taking several hours on most nights (insomnia), with final wakening described by parents as earlier in the morning than typical for her. Psychiatric symptoms included flat affect with possible hallucinations, characterized by behavior of attempting to bat things away, and loss of interest in previously enjoyable activities. She lost the abilities to help with dressing, toileting and to hold a pencil and write. She frequently lost the ability to remain engaged in a task, and was generally described by parents to appear to be “in a daze” for up to hours at a time. Neurological symptoms included tics and rhythmic motions, facial jerks, chorea-type movements, and head drop. She exhibited laughter at inappropriate times and an inability to stop eating.

### Evaluations

She was evaluated at an outside hospital at 3 weeks following symptom onset. Laboratory workup included cerebrospinal fluid (CSF) analyses and basic laboratory measures (complete blood count with differential, thyroid stimulating hormone, comprehensive metabolic panel, urinalysis, and urine drug screen). A prolonged EEG indicated slow background for age with higher amplitudes than expected. Brain MRI with and without contrast was normal.

She was evaluated at Duke University Medical Center (DUMC) 7 weeks after symptom onset. Evaluations included immunologic measures, repeat basic laboratory measures and repeat EEG. Lumbar puncture was not repeated. Long-term EEG was non-epileptic in nature with generalized diffuse slowing. Table 1 illustrates laboratory results, including immunologic laboratory measures, basic laboratory measures and cerebrospinal fluid (CSF) analyses. Anti-nuclear antibodies (ANA) were 1:40 (not considered clinically significant). Anti-streptolysin O was elevated at 469 (reference range ≤99), consistent with prior history of strep infection but not diagnostic of an active strep infection (had negative swab by PCP). All other laboratory results were within normal limits. No formal neuropsychological or cognitive assessments were completed as part of her clinical evaluation as the patient was too catatonic at her initial evaluation with us to complete any formal testing. No polysomnography was completed, as she was not noted to have any known risk factors for obstructive sleep apnea.

**Table 1:** Immunological evaluation of a patient with Down syndrome disintegrative disorder.

	**Result and reference range**
**Immunologic measures**	
Autoimmune encephalopathy panel (serum)	See Supplementary Table 1
Anti-cardiolipin antibody	Anti-cardiolipin IgG = 1 (≤ 15 units); IgM = 5 (≤ 14 units); IgA = 1 (≤ 14 units)
Angiotensin-converting enzyme	50 (22-108 U/L)
Anti-Beta-2-glycoprotein 1	IgG = 1 (≤ 20 units); IgM = 2 (≤ 20 units); IgA = 1 (≤ 20 units)
Von willebrand factor	89.0 (40.0–190.0%)
Lupus anticoagulant panel	See Supplementary Table 2
Anti-thyroglobulin antibodies	<1.8 (<4.0 IU/mL)
Anti-microsomal (thyroperoxidase) antibodies	0.9 (<9.0 IU/mL)
Anti-nuclear antibody screen	Positive (1:40 Speckled)
Anti-streptolysin O	469 (≤ 99 IU/mL)
**Basic laboratory measures**	
Complete blood count	See Supplementary Table 3
Sedimentation rate	2 (0–13 mm/h)
C-reactive protein	<0.02 (≤ 0.60 mg/dL)
Comprehensive metabolic panel	See Supplementary Table 4
Thyroid stimulating hormone	2.229 (0.200–4.500 IUI/mL)
**Cerebrospinal fluid analyses**	
Cell counts	See Supplementary Table 5
Glucose	61 (60–80 mg/dL)
Protein	34 (15–45 mg/dL)

### Treatment

**Figure 1:**
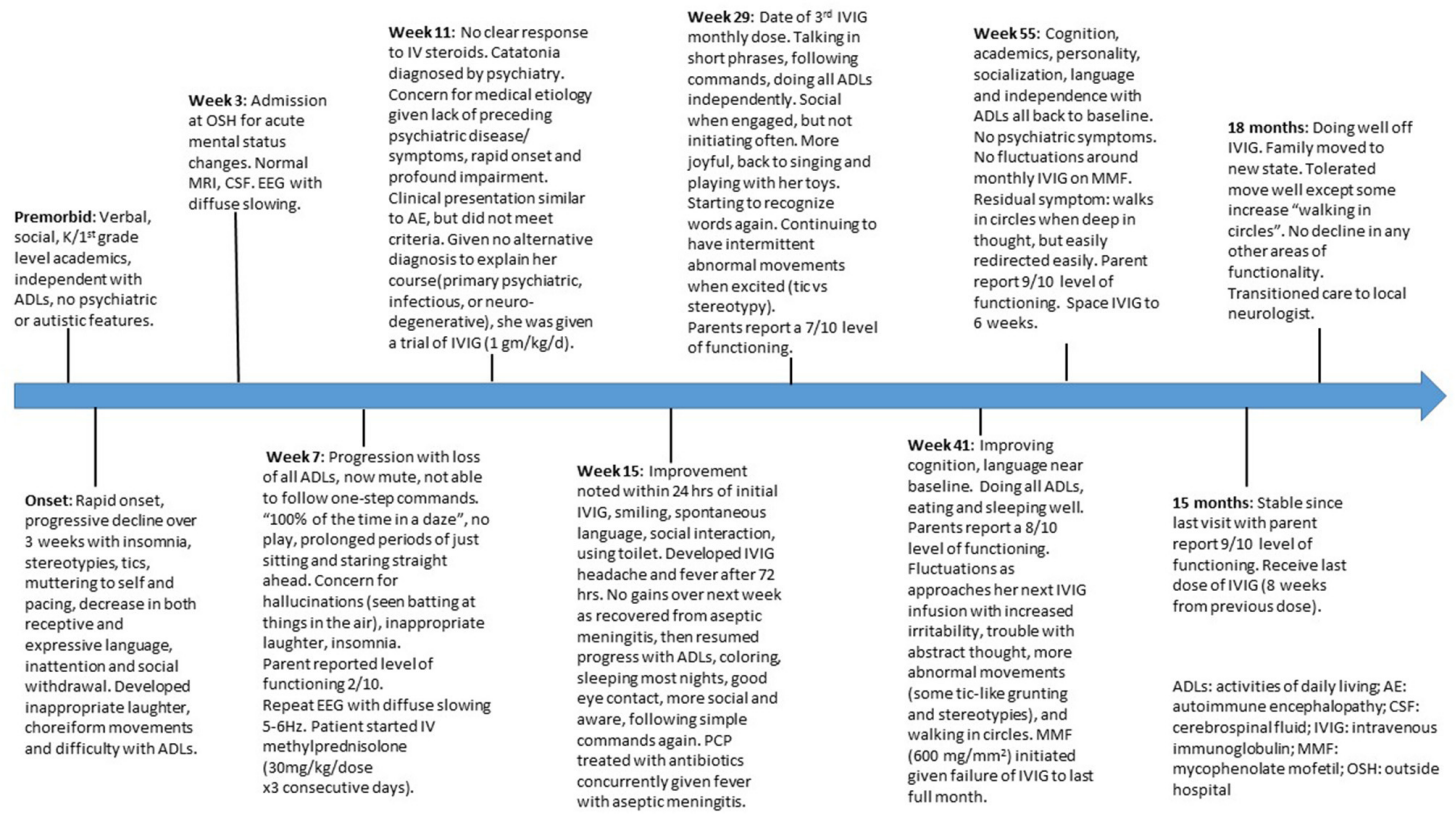
Timeline of treatment course.

A timeline of the treatment course is presented in Figure 1. About 2 months after onset of her initial symptoms, she was treated with 3 days of steroids (methylprednisolone, 30 mg/kg) with no clear improvement, but no further regression. She then received two consecutive days of both methylprednisolone (30 mg/kg/day) and intravenous immunoglobulin (IVIG) (1 g/kg/day) with notable improvement within 24 h. She developed aseptic meningitis post-IVIG with headache and fever. Her PCP started antibiotics due to fever and history of elevated strep titers. After recovering from the aseptic meningitis, she again started to improve. Within a few weeks, she regained some language, the ability to feed herself, the ability to follow simple commands, and had improved sleep and social interactions. There was a 1-month delay for her second planned monthly dose of IVIG due to insurance limitations. During this time she had some regression in her gains, before she again received monthly IVIG at 1 gm/kg/day in combination with methylprednisolone treatment (30 mg/kg/day) over 2 days for 1 month, and then monthly IVIG at 1 gm/kg. Her steroids were tapered to 10 mg/kg and maintained at this level with all IVIG doses as a pretreatment to reduce her IVIG side effects. She received this regimen for the next 8 months. Her parents noticed a pattern of increased tics, abnormal movements, and irritability in the week approaching the next monthly infusion. Given concerns this pattern indicated ongoing disease activity, she was started on mycophenolate mofetil (MMF) (600 mg/m^2^ twice daily) 7 months after initiation of IVIG. Complete blood counts and serum MMF levels were monitored. Within 3 months of initiation, she had no further regression as she approached her next dose and after 9 months of treatment, her cognitive function had returned to her pre-morbid state. IVIG treatments were then spaced to every 6 weeks for two treatments, then to every 8 weeks, completing a 12-month course of IVIG. Treatments with methylprednisolone/IVIG were then stopped while she continued on MMF. After her final treatment with methylprednisolone/IVIG, her care was transitioned to local providers with continued treatment with MMF. No additional medications were administered during the course of her treatment other than immunotherapy.

### Post-treatment Outcomes

Following her treatment across a period of 9 months, her catatonia resolved. She engaged in social activities, was smiling and interacting, playing with toys, and regained her ability to dress and toilet independently. She regained the ability to follow instructions and to make eye contact. Her features of autism resolved with speech returning to full sentences. She continued to have some residual tics with shoulder and arm movements and hand tapping, however. Her sleep showed significant improvement in overall sleep quality. Psychiatric symptoms resolved and she returned to engaging in enjoyable activities with no evidence for hallucinations. She showed significant improvement in cognition and mood. Her learning and memory deficits returned to her pre-morbid state with regained ability to recognize words. She began coloring and trying to write again. However, 3 months after the IVIG was stopped she had increased symptoms, which again resolved with repeat IVIG and slow taper off with no recurrence. She continued on MMF at that time. At the final follow up point 2.5 years after initial presentation, she was off IVIG and MMF with full recovery of speech, personality and learning abilities to her pre-morbid state, with only mild residual tics.

## Discussion

We describe a case of an individual with DSDD presenting with rapid-onset encephalopathy and catatonia who demonstrated marked improvement in symptoms following treatment with IVIG and steroids, despite lacking evidence of autoimmunity found through standard-of-care testing for autoimmune brain diseases. While the etiology of DSDD is unclear, an immune mediated process has been proposed as a possible explanation. This case suggests that immunotherapy (steroids, IVIG alone, or in combination with other agents including MMF) should be considered as a potential treatment option in individuals with rapid, profound decline consistent with DSDD, in whom other plausible etiologies have been excluded. In these patients, the absence of known autoantibodies or clear signs of inflammation should not be an absolute exclusion for treatment.

The differential for acute onset of neuropsychiatric symptoms are broad, and include autoimmune encephalitis (AE), PANS/PANDAS, infections, metabolic/genetic disease and primary psychiatric disease. Our patient had a clinical phenotype reminiscent of AE, with multiple domains of impairment (loss of language, cognitive decline/memory impairment, movement disorder, psychosis, progressing to catatonia), but her work up did not support an AE diagnosis as she had a normal brain MRI, lumbar puncture and CSF studies. PANS/PANDAS was also considered, especially given she had elevated strep titers. However, she did not have the clinical features required by the diagnostic criteria, as she had no obsessive-compulsive symptoms, anxiety, or restrictive eating (12). She did have tics, but these were mild and in the setting of several other movement abnormalities. While her strep titers were elevated, this is consistent with her prior history of strep (>9 months prior to illness onset), and the titers themselves are not considered diagnostic (12). In addition, she was treated with 4 weeks of antibiotics by her PCP with no clear improvements (improved when on IVIG, declined while still on antibiotics during delay for second dose of IVIG). The prior case series of individuals with DSDD with thyroid disease or other autoimmune disease showed improvement with immunotherapy, though they would not have met criteria for AE (8). We hypothesize some individuals with DSDD, especially those with a rapidly progressive presentation similar to AE, have an inflammatory or autoimmune etiology and may benefit from immunotherapy, regardless of whether or not they have detectable presence of autoantibodies.

Individuals with Down syndrome have been found to have proteomic differences associated with chronic immune dysregulation (13), which may be a risk factor for both auto-inflammation and encephalopathy (8). The individual in this case presentation required ongoing immunotherapy because her symptoms returned when the frequency of IVIG was decreased. This suggests that her IVIG truly had a therapeutic effect, rather than reflecting episodic fluctuations in symptom severity. It further suggests that an underlying immune dysregulation was responsible, given her improvements and ability to stop IVIG after adding MMF.

The therapeutic mechanisms of IVIG are complex; it may provide therapeutic benefit for both autoimmune and inflammatory diseases through multiple different processes (14). Other agents that may be used to decrease antibodies include MMF, methotrexate, rituximab and bortezomib. Electroconvulsive therapy (ECT) has historically been used to treat catatonia in the setting of multiple different psychiatric and neurological disorders (15), including in patients with Down syndrome (6, 7). ECT has been used in conjunction with immunotherapy in autoimmune encephalitis, most commonly reported in NMDA encephalitis. The mechanisms by which ECT are thought to work include enhancement of GABAergic functions, and in autoimmune encephalitis, potential down-regulation of immune activation with repetitive sessions of ECT (16). ECT has been found to be efficacious as a standard therapy for catatonia and may have potentially synergistic effects with other treatments such as lorazepam (7). Further, one of our previously reported patients had additional benefit when ECT was added to immunotherapy (6). ECT is associated with cardiovascular effects including dysrhythmias, but the effects are typically transient and generally resolve without adverse consequences (15). ECT may require special consideration in patients with Down syndrome given the higher prevalence of congenital heart defects.

It is notable that the current case presented at 8 years old, which is a younger age of onset than has typically been reported for DSDD. While the typical age of onset has been reported during adolescence and young adulthood, individuals with features consistent with DSDD have been reported between ages 4 and 30 (4). It is currently unknown how age of onset may relate to natural history or treatment outcomes for DSDD.

This patient and our four previously reported other patients did not meet diagnostic criteria for autoimmune encephalitis (8). We posit that they might have a condition analogous to other proposed immune mediated encephalopathies, such as Hashimoto encephalopathy but without the necessity of thyroid autoimmunity for the diagnosis. We recognize DSDD as a clinical syndrome representing a marked regression in skills across domains, and do not propose that all cases stem from a single etiology. For those who have had a thorough evaluation with no other clear etiology such as depression, trauma, or other medical condition, considering an immune mediated process and a trial of immunotherapy may be warranted, regardless of antibody status. If future studies are able to replicate the finding that some individuals with features of DSDD respond to immunotherapy, it may be appropriate to consider an alternative name for the diagnosis in these individuals, such as Down syndrome Autoimmune Encephalopathy.

## Data Availability Statement

The original contributions presented in the study are included in the article/Supplementary Material, further inquiries can be directed to the corresponding author/s.

## Ethics Statement

The studies involving human participants were reviewed and approved by Duke University Medical Center IRB. Written informed consent to participate in this study was provided by the participants' legal guardian/next of kin.

## Author Contributions

SH collected data from medical records, contributed to design of the study, and wrote the first draft of the manuscript. GW, PK, and HV contributed to the conception and design of the study. HV provided clinical evaluation and treatment for the patient. All authors contributed to manuscript revision, read and approved the submitted version.

## Conflict of Interest

The authors declare that the research was conducted in the absence of any commercial or financial relationships that could be construed as a potential conflict of interest.
