# Supplementary material for: Case Report: Improvement Following Immunotherapy in an Individual With Seronegative Down Syndrome Disintegrative Disorder
Source: Front Neurol. 2021 Mar 26;12:621637. doi: 10.3389/fneur.2021.621637 (PMC8032932; doi:10.3389/fneur.2021.621637)
Supplement: Supplementary file 1 [file Table_1.DOCX]

Supplementary Material

# Supplementary Table 1: Autoimmune encephalopathy panel (serum)

| Result name | Value (reference value) |
| --- | --- |
| NMDA-R Ab CBA | Negative (Negative) |
| Neuronal (V-G) K+ Channel Ab | 0.00 (<= 0.02 nmol/L) |
| LGI 1-IgG CBA | Negative (Negative) |
| CASPR2-IgG CBA | Negative (Negative) |
| GAD65 Ab Assay | 0.00 (<= 0.02 nmol/L) |
| GABA-B-R Ab CBA | Negative (Negative) |
| AMPA-R Ab CBA | Negative (Negative) |
| ANNA-1 | Negative (<1:240 titer) |
| ANNA-2 | Negative (<1:240 titer) |
| ANNA-3 | Negative (<1:240 titer) |
| AGNA-1 | Negative (<1:240 titer) |
| PCA-1 | Negative (<1:240 titer) |
| PCA-2 | Negative (<1:240 titer) |
| PCA-Tr | Negative (<1:240 titer) |
| Amphiphysin Ab | Negative (<1:240 titer) |
| N-type Calcium Channel Ab | 0.00 (<=0.03 nmol/L) |
| P/Q-Type Calcium Channel Ab | 0.00 (<=0.02 nmol/L) |
| Ach Receptor (Muscle) Binding Ab | 0.00 (<=0.02 nmol/L) |
| AChR Ganglionic Neuronal Ab | 0.00 (<=0.02 nmol/L) |
| CRMP-5-IgG | Negative (<1:240 titer) |

# Supplementary Table 2: Lupus anticoagulant panel

| Component | Value (reference range) |
| --- | --- |
| Prothrombin Time | 9.9 (9.5 - 13.1 sec) |
| Prothrombin INR | 0.9 (0.9 - 1.1) |
| Act Partial Thromboplastin Time | 21.3 (L) (26.8 - 37.1 sec) |
| Thrombin Clot Time | 18.8 (16.6 - 24.3 sec) |
| Dil Russell Viper Venom Screen | 0.79 (<1.20) |
| Lupus Anticoagulant Panel Interpretation | A lupus anticoagulant is NOT present. |

# Supplementary Table 3: Complete blood count

| Component | Value (reference range) |
| --- | --- |
| WBC | 4.2 (3.8 - 12.7 x10ˆ9/L) |
| Hemoglobin | 14.7 (11.4 - 15.5 g/dL) |
| Hematocrit | 41.8 (35.0 - 45.0 %) |
| Platelet Count /L | 230 (150 - 400 x10ˆ9/L) |
| MCV | 90 (77 - 95 fL) |
| MCH (Mean Corpuscular Hemoglobin) | 31.7 (25.0 - 33.0 pg) |
| MCHC | 35.2 (31.0 - 37.0 %) |
| RBC | 4.64 (3.80 - 5.50 x10ˆ12/L) |
| RDW-CV (Red Cell Distribution Width) | 13.2 (11.5 - 14.5 %) |
| NRBC (Nucleated Red Blood Cell Count) | 0.00 (0 x10ˆ9/L) |
| Nucleated RBC % | 0.0 % |
| MPV | 9.2 (7.2 - 11.7 fL) |
| Neutrophils | 2.1 (1.7 - 7.2 x10ˆ9/L) |
| Neutrophil % | 49.4 (39 - 65 %) |
| Lymphocyte Count | 1.7 (L) (1.8 - 4.8 x10ˆ9/L) |
| Lymphocyte % | 39.2 (27 - 50 %) |
| Monocyte Count | 0.4 (0.1 - 0.8 x10ˆ9/L) |
| Monocyte % | 9.3 (1 - 12 %) |
| Eosinophils | 0.03 (0 - 0.70 x10ˆ9/L) |
| Eosinophil % | 0.7 (0 - 9 %) |
| Basophils | 0.03 (0 - 0.20 x10ˆ9/L) |
| Basophil% | 0.7 (0 - 2 %) |
| Immature Granulocyte Count | 0.03 (<=0.06 x10ˆ9/L) |
| Immature Granulocyte % | 0.7 (<=0.7 %) |

# Supplementary Table 4: Comprehensive metabolic panel

| Component | Value (Reference range) |
| --- | --- |
| Sodium | 140 (136-143 mmol/L) |
| Potassium | 4.1 (3.5-5.0 mmol/L) |
| Chloride | 107 (98-110 mmol/L) |
| CO2 | 24 (22-30 mmol/L) |
| BUN | 17 (5-15 mg/dL) |
| Glucose | 74 (60-100 mg/dL) |
| Creatinine | 0.42 (0.40-0.90 mg/dL) |
| Calcium | 9.5 (8.5-11.0 mg/dL) |
| Total protein | 6.9 (6.0-8.0 g/dL) |
| Albumin | 4.4 (3.8-5.4 g/dL) |
| Total bilirubin | 0.4 (0.1-1.2 mg/dL) |
| Alkaline phosphatase | 200 (100-450 IU/L) |
| AST (SGOT) | 25 (15-50 IU/L) |
| ALT (SGPT) | 14 (15-50 IU/L) |
| Anion gap | 9 (4-14 mmol/L) |

# Supplementary Table 5: Cell counts (Cerebrospinal Fluid)

| Component | Value (Reference range) |
| --- | --- |
| Color | Colorless |
| Appearance | Clear |
| WBC | 0 (1-10/mm3) |
| RBC | 0 (0/mm3) |
